# The State of the Art of Plant Virus Research in China

**DOI:** 10.3390/v16101639

**Published:** 2024-10-21

**Authors:** Feng Li

**Affiliations:** College of Horticultural and Forestry Sciences, Huazhong Agricultural University, Wuhan 430070, China; chdlifeng@mail.hzau.edu.cn

Plant viruses impose serious threats to agriculture in China and worldwide [1]; to combat this, there is a large consortium of scientists working on basic plant virology and its applications in the field. In recent years, numerous important advancements have been achieved by Chinese scientists in major areas of plant virology, such as the discovery and analysis of new viruses in the field, the mechanisms of plant–virus interactions, and the development of virus detection technology. We gathered eleven papers on this topic, including two comprehensive reviews on the mechanisms of plant–virus interactions and nine research papers covering the above-mentioned areas.

The framework of the antiviral RNA silencing pathway has been established for approximately two decades; in this pathway, Dicer-like proteins (DCL2 and 4) perceive invading viral double-stranded RNA (dsRNA) and process it into viral siRNAs (vsiRNA), the effector protein Argonautes (AGO1 and 2) loads vsiRNAs and slices viral RNA, and RNA-dependent RNA polymerases (RDR1 and 6) amplify vsiRNAs and promote antiviral silencing [2,3]. Jin et al. reviewed a large volume of both the classic and the recent literature on the mechanisms of antiviral RNA silencing and its suppression by various viruses, adding new players from both plants and viruses, to form an updated comprehensive picture of how plants fight against viruses using vsiRNA and how viruses counteract this defense with viral suppressor of RNA silencing (VSR) [4]. The review by Zhang et al. focused on specific interactions between plants and geminiviruses. Different defense mechanisms, including antiviral silencing, protein kinase-mediated immunity, effector-triggered immunity, autophagy, etc., were covered in this review, which provided a comprehensive picture on how plants fight against geminiviruses [5].

As viruses continuously evolve, new viral diseases constantly emerge. The identification and characterization of viruses from diseased plants are important starting points for both basic research and the control of viral diseases. Nodaviruses are usually considered to be animal viruses. Interestingly, Xie et al. identified two novel noda-like viruses in rice plants that were showing dwarfing symptoms, and demonstrated that RNA1 from one of these viruses can replicate in both plant and insect cells [6]. Bunyaviruses have been reported to cause disease in plants, but they have not been found in rice before. Wang et al. identified a bunya-like virus in rice plants with dwarfing symptoms, and a sequence analysis showed that it was related to the viruses from the family *Discoviridae* [7]. Sichuan is an important kiwifruit production area in China and viruses cause important yield loss in this crop. Shang et al. studied the occurrence and population structure of the major kiwifruit viruses, including Actinidia virus A, Actinidia virus B, Actinidia chlorotic ringspot-associated virus, and cucumber mosaic virus, in Sichuan province [8]. He et al. conducted codon usage analysis on three potyviruses infecting Narcissus and found that the codon usage biases of these viruses are similar and are mainly influenced by natural selection [9].

The detection and quantification of the virus’ and host’s gene expressions in the infected samples are important for studying the mechanisms of plant–virus interactions. The development of cost-effective, reliable, and sensitive viral and host gene detection methods is essential for the advancement of research in plant–virus interactions. Zhang et al. developed an simple-easy-to-use Northern blot method using vertical electrophoresis apparatus, which was originally used for Western blot analysis and T4 DNA polymerase reaction-based probe labeling. This method achieved cost-effective and highly sensitive detection of viral transcripts [10]. This probe labeling was also suitable for dot blots, which can simultaneously monitor viral RNA levels in large amounts of RNA samples [11]. Plum pox virus (PPV), also known as Sharka disease, affects stone fruit trees and has the potential to result in enormous economic losses worldwide. In response to the need to monitor PPV, Guo et al. generated two highly sensitive and specific anti-Plum pox virus monoclonal antibodies, which facilitated the development of a dot enzyme-linked immunosorbent assay (dotELISA) and a colloidal gold immunochromatographic strip (CGICS) assay [12]. Real-time quantitative PCR is a commonly used technique for studying gene expression levels, as it has high sensitivity. However, it is highly dependent on the stability of the reference genes. Dong et al. analyzed the stability of a number of reference genes during Ilarvirus infection and found that the combined use of *EF1α* and *F-BOX* gave the best normalization result [13].

Translation initiation factors are reported to play both positive and negative roles in viral mRNA translation [14], and recessive resistance against many viruses conferred by the mutation of various translation initiation factors has been reported in many plant–virus interaction cases [15]. In rice, natural alleles with point mutations in both eIF4G and eIFiso4G were reported to confer resistance against rice yellow mottle virus and rice tungro spherical virus, respectively [15]. Wang et al. generated a CRISPR mutant of eIF4G with 3 nucleotide deletion in its 5’ part of the open reading frame [16]. This mutation reduced eIF4G transcript levels and enhanced tolerance to rice black-streaked dwarf virus (RBSDV) but did not cause any developmental phenotypes; thus, it provided useful resources for developing RBSDV-resistant varieties [16]. A recent study showed that eIFiso4G is a pattern-triggered immunity-activating factor that promotes plant stress-responsive mRNA translation [17]. Viruses have obviously learned to exploit host stress-response factors for its only translation. Interestingly, Zhang et al. reported a similar host factor, SR4N, which participates in effector-triggered immunity but is also exploited by multiple viruses to promote viral accumulation [18]. Plant cellular membrane structures provide essential support to viral replication [19,20]. Targeting viral RNA-dependent RNA polymerase (RdRp) in a proper membrane structure is important for successful viral replication. Jiang et al. showed that the first α-helix of the methyltransferase (MET) domain is required for multimerization and the endoplasmic reticulum membrane association’s with potato virus X RdRp, providing new insights into the formation of the viral replication complex [21].

We hope that the review papers in this Special Issue provide an up-to-date picture of the mechanisms of plant–virus interactions, that the advancements in technique facilitate further studies in virology and other related fields, and that the surveys of viruses provide a good basis for developing strategies to control newly emerged viral diseases.
